# Is tool-making knowledge robust over time and across problems?

**DOI:** 10.3389/fpsyg.2014.01395

**Published:** 2014-12-04

**Authors:** Sarah R. Beck, Nicola Cutting, Ian A. Apperly, Zoe Demery, Leila Iliffe, Sonia Rishi, Jackie Chappell

**Affiliations:** ^1^School of Psychology, University of BirminghamBirmingham, UK; ^2^School of Biosciences, University of BirminghamBirmingham, UK

**Keywords:** tool use, innovation, problem solving, cognitive development, pedagogy, social learning, analogy

## Abstract

In three studies, we explored the retention and transfer of tool-making knowledge, learnt from an adult demonstration, to other temporal and task contexts. All studies used a variation of a task in which children had to make a hook tool to retrieve a bucket from a tall transparent tube. Children who failed to innovate the hook tool independently saw a demonstration. In Study 1, we tested children aged 4–6 years (*N* = 53) who had seen the original demonstration 3 months earlier. Performance was excellent at the second time, indicating that children’s knowledge was retained over the 3 month period. In Studies 2 and 3 we explored transfer of the new knowledge to other tasks. In Study 2, children were given two variants of the apparatus that differed in surface characteristics (e.g., shape and color). Participants generalized their knowledge to these new apparatuses even though the new pipecleaner also differed in size and color. Five- to 6-year-olds (*N* = 22) almost always transferred their knowledge to problems where the same tool had to be made. Younger, 3- to 5-year-olds’ (*N* = 46), performance was more variable. In Study 3, 4- to 7-year-olds (*N* = 146) saw a demonstration of hook making with a pipecleaner, but then had to make a tool by combining pieces of wooden dowel (or vice versa: original training on dowel, transfer to pipecleaner). Children did not transfer their tool-making knowledge to the new material. Children retained tool-making knowledge over time and transferred their knowledge to new situations in which they needed to make a similar tool from similar materials, but not different materials. We concluded that children’s ability to use tool-making knowledge in novel situations is likely to depend on memory and analogical reasoning, with the latter continuing to develop during middle childhood.

## INTRODUCTION

The tools that facilitate human society differ in two ways from the majority of non-human animal tools. First, the number of different tools that we use far surpasses the number used by any other non-human species. Second, almost all of the tools that humans use are made not found. Although some non-human species make tools, including some corvids such as New Caledonian crows (Hunt and Gray, 2004) and rooks (Bird and Emery, 2009), and chimpanzees (Boesch and Boesch, 1990), this is relatively unusual (Shumaker et al., 2011).

Despite being experts when learning how to use tools from others (e.g., Hopper et al., 2010), recent studies have shown a remarkable limit to children’s ability to make tools. Children under 8 years are very unlikely to bend a piece of wire in to a hook spontaneously to retrieve a reward-holding bucket from a tall transparent tube. This is true of samples of children from Western, technology-rich communities in the UK (Beck et al., 2011) and children from the Bushman communities who, compared to Western children, have “a lack of reliance on direct instruction in learning and greater exposure to needing to make artifacts that are played with” (Nielsen et al., 2014, p. 386).

Children’s tool making has been investigated using a paradigm adapted from the non-human animal literature. A bucket baited with a reward [food for corvid participants (Weir et al., 2002), stickers for human children (Beck et al., 2011)] was placed at the bottom of a tall transparent tube. The participant had a straight piece of wire (a pipecleaner for children) that s/he needed to bend into a hook in order to retrieve the bucket and claim the reward. Making a hook tool independently (termed tool innovation) was extremely difficult: very few 3- to 5-year-olds succeeded, increasing to only around 50% of children by the age of 7. Children who failed to retrieve the bucket progressed to a second phase where the experimenter demonstrated how to make a tool using an identical pipecleaner. The pattern of performance reversed. Almost all children, even those as young as 3 years old, followed the experimenter’s demonstration to make their own hook, which, they then immediately used appropriately to retrieve the bucket (Beck et al., 2011). Success after the demonstration was termed tool manufacture.

This dissociation in performance between the two forms of tool making (creating novel tools and benefitting from shared information) has been replicated in several further studies. Children’s difficulties making novel tools persist when they have the chance to use the materials before encountering the tool-making problem (Beck et al., 2011). They are also seen on different tasks. For example, Cutting et al. (2011) presented children with a problem where they had to unbend a folded pipecleaner to make a long straight tool to push a ball from a tube. Children’s tool innovation was not promoted by explicit instructions to “make something” (Cutting et al., 2011) nor suggestions to try a different strategy (Chappell et al., 2013). Yet in all these studies children found it easy to make a tool given a demonstration and (almost always without instruction) used the new tool to solve the task. We can say with some confidence that tool innovation is difficult for young children, whereas learning from others how to make new tools (tool manufacture) is easy.

Humans are particularly well adapted for social sharing of information especially via pedagogy (see Csibra and Gergely, 2009). We know that young children are experts at learning from others how to use tools^1^ (e.g., Hopper et al., 2010). Our goal in this paper is to extend this finding to tool making. Tool-making knowledge is particularly important to share because it provides novel technology to solve problems.

On a community scale this sharing is important because as long as one has good processes for sharing information, the work of just a few innovators (or even lucky accidents and slow collaborations) can be preserved. However, for this sharing to be effective, the recipients need to do more than imitate others’ tool making in the moment. The knowledge acquired from observations about how to make a tool needs to be robust enough to generalize out of the immediate context. Tool-making knowledge would be most useful if it were preserved over long periods of time and could be applied to new instantiations of similar problems. Indeed, even with many innovators and a strong tendency to learn from others, if the knowledge acquired is not robust, then the spread of novel tool making will be severely restricted.

Thinking first about the robustness of children’s knowledge over time, there already exists a body of work on very young children’s memory for information, especially actions, that they learn from others. Meltzoff (1985) demonstrated that infants at 14 months could sometimes reproduce behavior (turning on a light with the head) after 24 h, terming this deferred imitation. Herbert et al. (2006) showed that even younger children, 9-month-olds (but not 6-month-olds), could remember to press a button on a toy 24 h after they had seen a demonstration. As children get older they are able to retain information about actions and objects over a longer period of time: Herbert and Hayne (2000a) demonstrated a three step process to 24-month-olds who reproduced an average of 2 of the three steps 8 weeks later. Relevant for our interest in tool *making*, this study also involved construction of an object as children put together three components to make a rattle. Bauer’s (2002) work (see for review) gives further evidence that children under two can recall multi-step sequences over long periods of time, with 20-month-olds remembering the order of some sequences up to 12 months after exposure (Bauer et al., 2000).

Looking specifically at children using tools to solve problems, their memory for actions is impressive: Óturai et al. (2012) showed that 18-month-olds could reproduce the most functional aspects of how to use a tool 2 weeks after the original observation. Simpson and Riggs (2011) showed that 3-year-olds not only remembered how to use a tool to open a puzzle box after a week, but were selective in the information they reproduced. When given the chance to solve the puzzle immediately after a demonstration, 3-year-olds reproduced both functionally relevant and irrelevant actions. After a week, irrelevant actions had largely disappeared.

For knowledge learnt from others to be potentially useful in spreading innovation it needs to be robust across new instantiations of similar problems. Some authors have looked to see whether changes in the context affect children’s reproduction of behavior. Patel et al. (2013) tested children in their first year of life using a very simple action – removing a mitten from a puppet and shaking it to sound a bell. They found that 6-month-olds consistently reproduced the action 24 h later only if tested in the same context (the same room with the same music playing). By 12 months of age children could generalize the behavior to new contexts where both of these elements were changed. Herbert and Hayne (2000b) examined how changes to the objects involved in the actions might influence children’s retrieval. Using the same rattle-making task as Herbert and Hayne (2000a), 30-month-olds saw an experimenter construct a rattle using three items on day 1 but were presented with rather different objects (e.g., a transparent cup instead of an opaque red ball) after 24 h. Thirty-month-olds were able to construct a rattle using the new stimuli set.

Clearly there is evidence that very young children are able to reproduce information taught to them by others, generalizing this across time and across new instantiations of similar problems. Our interest in this paper was to extend this to tool-making in a problem-solving context. Tool making is a new domain for research in children’s tool use and has already revealed the surprising finding that tool innovation is exceptionally difficult for young children. As well as wanting to know more about children’s developing competence in this domain, the general topic is itself important to understand because the generalization of innovations about how to make tools is so important for our tool-rich culture. Are even very young children able to deploy tool-making knowledge in a way that would support the spread of innovations?

In three studies we explored the robustness of tool-making knowledge gained by children from others. Each study focussed on one aspect of robust generalization: retention over time (Study 1) and transfer across problems (Studies 2 and 3). In these, the first studies of children’s learnt knowledge of tool-making, we aimed to expand our understanding of children’s tool making, to explore in principle whether their knowledge is sufficiently robust that it could transmit innovation through a culture and to illustrate new topics for further research in this field.

## STUDY 1

We investigated the first way in which tool-making learning might be robust: do children retain knowledge over time about how to make a simple tool that has been demonstrated to them. Our study was opportunistic. We had the chance to return to a school where we had previously tested 4- to 6-year-olds after a period of 3 months. In the original testing session at Time 1 (T1), children were presented with the transparent tube task with a straight pipecleaner and given a minute to retrieve the bucket (the original T1 data are reported in Cutting et al., 2014). In line with previous findings, innovation performance was poor, but most children succeeded after the experimenter demonstrated how to make (but not use) a hook. At Time 2 (T2) 3 months later, we gave children the possibility of demonstrating retention, i.e., the same experimenter re-presented children with the same apparatus. Our question was whether children would solve the task at T2 at higher levels of success than at T1.

In the original study, we had explored how the adult demonstration aided children in learning how to make a tool by using a two-stage demonstration. Children who failed to innovate their own tool were first shown simply a pre-made hook (made from an identical pipecleaner), but not how to make it. If, after a further 30 s, children were still unsuccessful, the experimenter demonstrated how to make the tool: bending an identical pipecleaner into an appropriately sized hook in full view of the child. Would the robustness of children’s knowledge of how to make a simple tool, demonstrated at Time 2, be influenced by how they learnt the technique at Time 1? One possibility is that children who were shown an end-state demonstration but figured out the means to make the tool themselves may have better knowledge. On the other hand, if children needed the bending demonstration to succeed, then they would have had more exposure to hooks and hook making at T1, which may lead to more resilient memories. A third possibility was that children’s memories are relatively insensitive to how knowledge about tool making is gained.

### METHOD

#### Participants

Fifty-eight children (29 girls) participated in the original study. Four of them did not participate at T2 (n.b. performance at T1 by these children was distributed across the different categories: 1 solved the task after the endstate demonstration, 1 after the bending demonstration, 2 failed to solve the task). The children who participated at T2 were 28 younger children (16 girls) aged 52–64 months, mean 57 months, and 25 older children (11 girls) aged 65–76 months, mean 71 months. Age groups were based on school class. Children were recruited from and tested at a school serving a predominantly working class white population in the UK.

#### Materials

**FIGURE 1:**
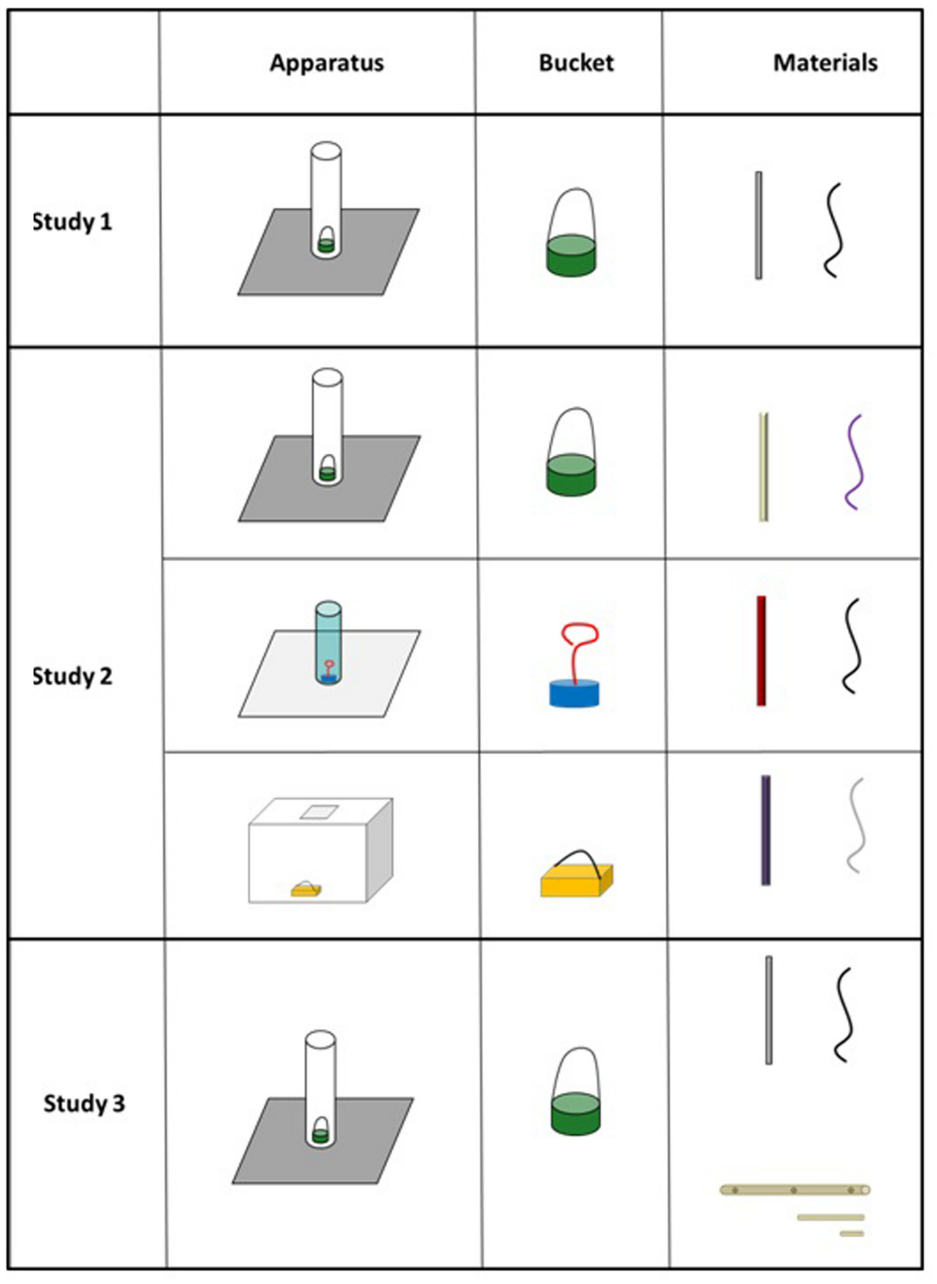
**Apparatus and materials used in the three studies**.

We used a tall transparent tube (22 cm tall with a 4 cm opening) attached to a board (35 cm × 21 cm, to prevent it being turned over), a straight white pipecleaner (29 cm), and a piece of black string (29 cm). As in previous studies, the string was included as a distractor and was not needed to solve the task. A small bucket was placed in the bottom of the tube containing a sticker (see **Figure 1**). Where a demonstration was necessary, the experimenter used a second identical pipecleaner.

#### Procedure

The procedures for the studies reported in this paper were approved by the University of Birmingham, UK, STEM Ethical Review Committee.

At both times children were tested in a quiet area away outside the classroom by a female experimenter (NC). They were shown the apparatus, pre-baited with the sticker and told, “If you can get the sticker out of here you can keep it.” They were given one minute to solve the task with the experimenter giving only neutral prompts if necessary, e.g., ‘you can try these things.’ If after one minute children failed to retrieve the bucket, the experimenter instructed them to stop and put down the materials. She then said ‘look at this’ and showed the child a hook made from an identical pipecleaner (target-tool demonstration). Children did not see the hook being made. Following this demonstration, children had a further 30 s to try to solve the task. If they had still not retrieved the bucket, the experimenter asked them to stop and put down the materials and then said ‘watch this.’ She demonstrated bending an identical straight pipecleaner into a hook (tool-creation demonstration). Children had a final minute to solve the task, with any who failed after this third phase being assisted by the experimenter. Children participated in the same procedure at T1 and T2 (3 months after T1). N.b. at T1 children were allocated to one of two conditions. One condition saw a warm-up phase where the materials were manipulated by the experimenter and the child. As this made no difference to tool-making success at T1 or T2, we do not consider this any further.

#### Analysis strategy

In the studies reported here we used the following non-parametric statistical analyses. When comparing between groups of children we used Pearson’s Chi Square test (with a continuity correction) where possible and Fisher’s Exact Test when the expected values were too small. When comparing performance of the same individuals on two measures we used McNemar tests. When exploring the relation between individuals’ performance on two measures we used Chi Square or Fisher’s Exact Tests. In this case we cross tabulated performance on the first measure (rows) against performance on the second measure (columns) allowing us to look for contingency between the two performances.

Because our analyses involved multiple comparisons of our data we made Bonferroni corrections based on the maximum number of planned *post hoc* comparisons in the main experimental design (i.e., we considered the most comparisons we would need to test our hypotheses, but we did not include every logically possible test – e.g., comparing age group 1 on trial A with age group 2 on trial B). We reported *p* values of <0.05 as trends.

### RESULTS AND DISCUSSION

In Study 1, we identified four planned *post hoc* comparisons [Success at each time compared between age groups (2) and comparison between T1 and T2 for each age (2)]. We report *p-*values <0.013 as significant.

Gender had no effect on children’s success (lowest *p* > 0.999) and so data from boys and girls were combined.

In Cutting et al. (2014) we reported children’s poor performance innovating a hook tool before any demonstration. We reproduce those data here for comparison purposes, see **Table 1**. Overall, it is clear that children were far more successful at making a hook tool before seeing a demonstrate on at T2 than T1: none of the younger children succeeded at T1, compared to 71% at T2; 16% of the older children made a hook without an adult demonstration at T1, 68% did so at T2. A McNemar test confirmed this difference in the older group: 14 children who failed at T1 succeeded at T2 and only one child showed the reverse pattern, *p* < 0.001. As no child succeeded at T1 in the younger group, we did not compute a statistical test.

**Table 1:** Success on hook making task at T1 and T2, Study 1.

Time 1		Time 2				% of children participating at this stage in T1 who succeeded (cumulative % of children passing by this stage)
		Pre-demonstration (innovation)	Target-tool demo	Tool-creation demo	Never succeeded
Younger children (4–5 years)	Pre-demonstration (innovation)	–	–	–	–	0% (0%)
	Target-tool demo	8	1	–	–	32% (32%)
	Tool-creation demo	6	3	1	–	53% (68%)
	Never succeeded	6	1	2	–	

Older children (5–6 years)	Pre-demonstration (innovation)	3	1	–	–	16% (16%)
	Target-tool demo	7	2	–	–	43% (52%)
	Tool-creation demo	4	2	–	–	50% (76%)
	Never succeeded	3	3	–	–	

% of children participating at this stage in T2 who succeeded (cumulative % of children passing by this stage)	Younger	71% (71%)	63% (89%)	100% (100%)		


	Older	68% (68%)	100% (100%)	–		

There was no difference between the younger and older children’s performance at Time 1 (*p* = 0.112) or Time 2 (*p* = 0.585).

We considered whether learning experience at T1 influenced performance at T2. Of the four children who passed without a demonstration at T1, three repeated this success at T2. Using Fishers’ Exact tests we made statistical comparisons between the larger groups of children who saw just the endstate demonstration and those who saw the endstate plus the bending demonstrations. There was no difference in likelihood of pre-demonstration success at T2 based on the demonstrations seen at T1: younger children *N* = 19, *p* = 0.303, older children *N* = 15, *p* < 0.999.

Our opportunistic study prevents us making a causal claim about the effect of seeing a demonstration at T1 on success at T2, as we did not have a control group of children at T2 who were not exposed to a demonstration at T1. However, the evidence strongly points to the conclusion that children who have learnt how to make a simple tool following a demonstration retain this knowledge for a substantial period of time (at least 3 months). Despite being 3 months older than they were at T1, naïve children at the age tested in T2 would be expected to struggle to solve the task without a demonstration. In a previous study, 7% of 4- to 5-year-olds and 36% of 5- to 6-year-olds succeeded spontaneously (Beck et al., 2011), compared to 71 and 68% in this study. Furthermore, within this study the younger children at T2 are still substantially younger than the older children at T1; the latter performed relatively poorly pre-demonstration (16% success) while 71% of the younger sample at T2 made a hook.

Our evidence suggests that when children learn how to make a tool by watching another this knowledge is robust, in at least one sense: most children retain this knowledge and are able to draw on it after a relatively long period of time.

## STUDY 2

In Study 2, we began our investigation of whether tool-making knowledge would transfer out of the immediate context. New instantiations of problems that require the same solution are likely to differ in surface characteristics. However, this cannot confuse the novice tool maker. She needs to recognize when a tool she already has in her repertoire could be useful.

There are two ways in which the knowledge gained from the tool-making demonstration might transfer to other contexts. First, children may learn something general about the possibility of making tools. They may be ‘primed’ for innovation. In this case, we would expect children to succeed when given tasks that required making simple tools other than hooks. In fact, we already know that this is not the case. In Cutting et al.’s (2011) study, children were sequentially presented with two different problems: the hook-making vertical tube task and a horizontal tube task where a bent pipecleaner had to be straightened to make a long tool to push a ball from the tube. Tasks were counterbalanced and there was no effect on success on the second task, having been shown how to make a tool to solve the first task. Making a tool following a demonstration did not lead to a general tendency for tool innovation. In Studies 2 and 3, having shown children a demonstration of how to make a tool (if needed), we then presented them with a similar problem that differed in surface characteristics. In Study 2 we varied the color, size and shape of the tube, and the color and size of the tool-making materials (pipecleaner and string).

### METHOD

#### Participants

Sixty-nine children participated in this study. One further 4-year-old child chose to withdraw midway through the study and his data are not presented here. Based on school class children were treated as a 3- to 4-year-old group, *N* = 20, 10 girls, mean age 46 months, range 40–53; a 4- to 5-year-old group, *N* = 26, 12 girls, mean age 61, range 55–66; and a 5- to 6-year-old group, *N* = 23, 11 girls, mean age 72, range 67–77. Children were recruited from and tested at a school serving a predominantly working-class black population in the UK.

#### Materials

We used the original tube (22 cm tall with 4 cm opening) with a cream colored 29 cm pipecleaner, a 29 cm length of purple string, a round green bucket, and cartoon character stickers. In the new versions of the task we used (1) a shorter green tube (17 cm tall with a 4.5 cm opening), red metallic 29 cm pipecleaner, a length of black string, a round blue bucket with a ‘hoop’ handle, and ladybug stickers and (2) a cuboid clear transparent box (20 cm square with a 4 cm opening), a 29cm purple pipecleaner, a length of silver colored string, a square yellow bucket and star stickers (see **Figure 1**).

#### Procedure

Children were tested in a quiet area away from the rest of the class by a female experimenter (ZD). There were three different versions of the task, each with distinct apparatus and tool-making materials. For each task, children were presented with the pre-baited apparatus and told ‘If you can get the sticker out of here you can keep it.’ Children were given 1 min to attempt to retrieve the bucket. If they did so, they were given the sticker and progressed to the next version of the task. If they did not retrieve the bucket independently, the experimenter asked them to stop and put down their materials and watch her. She took an identical pipecleaner to the one available on that task and demonstrated how to bend it in to a hook (tool-creation demonstration). Children were then given 30 s to try to retrieve the sticker. Unlike Study 1, we used a single stage demonstration to simplify analysis and to reduce the length of time taken by the study. Each child participated in all three versions of the task: the order was counterbalanced between children. After the first and second versions of the task the apparatus was cleared away before the new task was produced. After the third version, the child was thanked and returned to the classroom with his/her stickers.

### RESULTS AND DISCUSSION

In Study 2, we identified 18 planned *post hoc* comparisons [comparison of each age group’s performance on pairs of trials (9), comparison between pairs of age groups on each trial (9)]. We report *p*-values <0.003 as significant in our *post hoc* analyses.

Gender had no effect on children’s success (lowest *p* = 0.407) and so data from boys and girls were combined.

**FIGURE 2:**
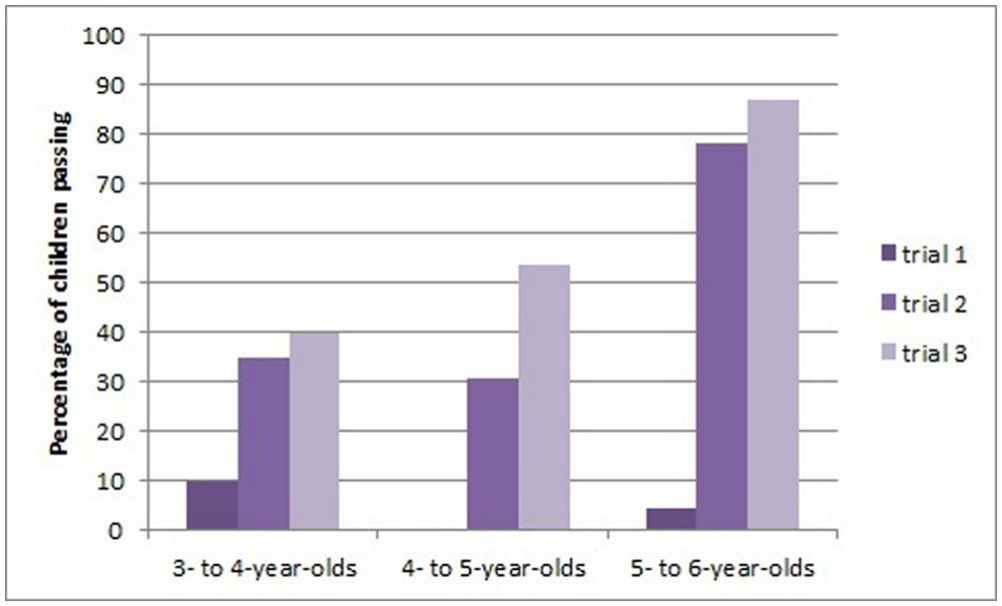
**Performance on each trial before demonstration in Study 2**.

We coded whether children bent the pipecleaner into a hook and used it successfully to retrieve the bucket from the tube before the demonstration for that trial. These results are shown in **Figure 2**. Including all children, regardless of age group, McNemar tests showed that performance was better on trial 2 compared to trial 1 (30 passed trial 2 only, none passed trial 1 only, *p* < 0.001) and better on trial 3 compared to trial 1 (40 passed trial 3 only, 1 passed trial 1 only, *p* < 0.001). There was no significant difference between trials 2 and 3 (*p* = 0.093).

We explored these patterns for each age group separately. For the oldest 5- to 6-year-olds performance on trial 2 was better than trial 1 (17 passed only the second compared to 0 who passed only the first, *p* < 0.001) and performance on trial 3 was better than trial 1 (19 passed only trial 3 compared to 0, *p* < 0.001). There was no improvement between trials 2 and 3 (two children passed only trial 2 and 4 passed only trial 3, *p* = 0.687). For the 4- to 5-year-olds we were not able to run statistical tests involving the first trial as the data were uniform: no child passed the first but not the second trial and eight showed the reverse and no child passed the first trial but not the third and 12 showed the reverse. There was no significant improvement between the second and third trials *p* = 0.070 (one child passed only trial 2 and 7 children passed only trial 3). For the youngest children (3- to 4-year-olds) there were no significant differences between trials. (lowest *p* = 0.063). However, the pattern of performance shown in **Figure 2** for both 3- to 4- and 4- to 5-year-olds makes us reticent to assert positively that there is no improvement in performance.

We made comparisons between the age groups on the different trials. On trial 1 only 3 of 69 children succeeded, so it was not possible to conduct a statistical test. However, on trials 2 and 3 Chi Square Tests revealed that performance differed by age group, trial 2 χ*^2^* (df = 2, *N* = 69) = 12.89, *p* = 0.002 and trial 3 χ*^2^* (df = 2, *N* = 69) = 10.77, *p* = 0.005. *Post hoc* Chi Square tests comparing between age groups showed trends to significance that the 5- to 6-year-olds performance better than both younger age groups on the later trials (*p-*values ranged from 0.002 to 0.028), whereas performance by the younger two age groups did not differ (lowest *p* = 0.526).

**FIGURE 3:**
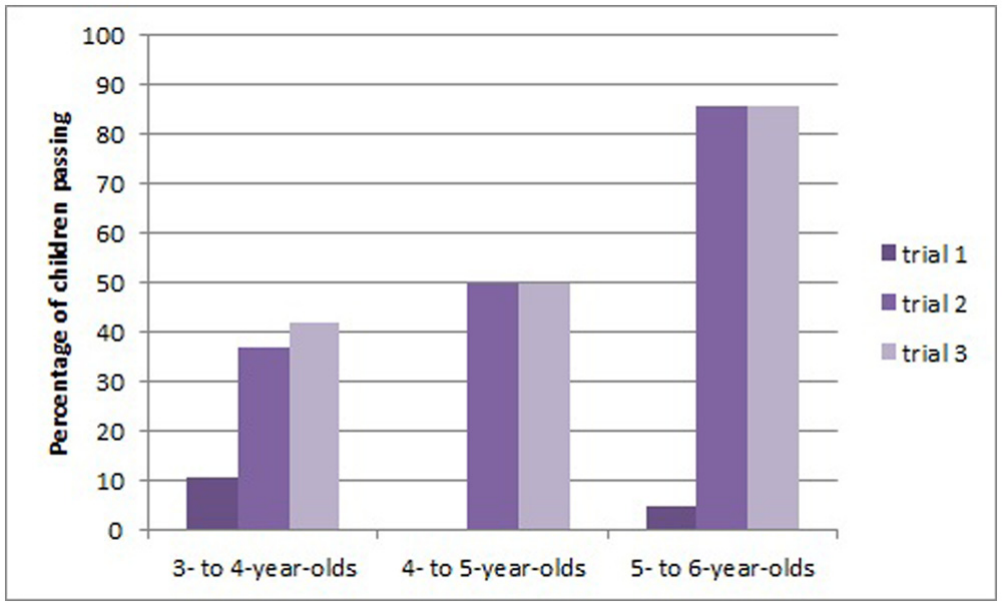
**Performance on each trial before demonstration in Study 2, reduced sample of children who had made a hook or seen a demonstration of hook making**.

However, these data are relatively conservative as they include all children in the comparisons, even some who did not see a hook-making demonstration before progressing to the next trial. One unanticipated problem was that some children managed to retrieve the bucket from the tube using a straight pipecleaner using a dragging or levering technique. This was a particular problem with the new green tube apparatus, which had short straight sides. Eight children did this with the green apparatus, four with the clear tube, and four with the box apparatus. Although these children were not counted as successfully making a hook, they did not then see a hook-making demonstration, but progressed to the second and third trials. Furthermore, on the second and third trials some children bent the pipecleaner into a hook shape, but then did not successfully retrieve the bucket. Perhaps it is not surprising that having seen a demonstration of bending for the first apparatus some children went on to make a hook, which did not have appropriate dimensions for the novel apparatus they were then presented with. Indeed, by changing the surface features of the tasks, we may also have inadvertently made the task more challenging in terms of technical skill. To make a less conservative estimate of children’s transfer to similar tasks, we excluded the children who solved the task without making a tool in trial 1. We also excluded data from four children who made a non-functional hook in trial 2 but did not then receive a demonstration of appropriate hook-bending. Thus, all children in the reduced sample (*N* = 54) had either solved the task by making a functional hook or seen a demonstration from the experimenter on failing trial 1 or 2. Using this reduced sample the pattern of results did not change from the previous analysis, apart from the comparison between the older two age groups on trials 2 and 3, which were no longer a trend (*p* = 0.053 in both tests). **Figure 3** shows the proportion of success in the reduced sample.

We explored whether individual children’s performance on the three trials was related. As performance was near floor on trial 1 we were not able to include this in our statistical analysis (n.b. three children solved the task spontaneously on trial 1. Of these two went on to solve trial 2 and 3 and the third child solved trial 2 but made an inappropriately sized hook on trial 3 that was non-functional). We used Chi Square to look for relations between performance on trials 2 and 3. There was a relation between these trials when all children were included, χ^2^(1,69) = 7.145, *p* = 0.008 (reduced sample χ^2^(1,54) = 7.891, *p* = 0.005). As children in the younger groups reached around 50% correct on the latter two trials, we also ran Chi Square tests excluding the oldest age group to see if performance on these trials was related. This was not the case, χ^2^(1,46) = 2.145, *p* = 0.143 (reduced sample, χ^2^(1,33) = 2.284, *p* = 0.131.

Overall, we draw two conclusions from this study. Children at all ages showed some ability to transfer knowledge to novel instantiations of a similar problem with different surface characteristics. This effect was strongest in 5- to 6-year-olds who went from near floor performance to near ceiling (from 5 to 86% success). Younger children’s (3- to 5-year-olds) transfer was less convincing. Their near floor performance increased to around 50% success, although was not shown to be a significant difference.

## STUDY 3

Study 2 demonstrated that children show some ability to transfer knowledge they gain from one problem to another similar problem which differs only on surface characteristics. In Study 3 we further explored children’s ability to transfer knowledge from one problem to another. Children were presented twice with the same tube apparatus. Each attempt at the task required them to make a hook tool, but we altered the means and materials by which they could make one. On one presentation children were required to bend a pipecleaner into a hook as per the previous studies. On the other presentation children were required to make a hook tool by adding pieces of dowel together to make a wooden hook (see Cutting, unpublished). This study was designed to investigate whether knowledge of making a hook tool would transfer to new materials and methods of tool making. We know that children do not transfer general tool-making knowledge when they need to make two different tools (Cutting et al., 2011), but can they generalize knowledge of a specific tool, a hook, to a new tool-making problem?

### METHOD

#### Participants

One hundred and forty six children participated in this study. Based on school class children represented a 4- to 5-year-old group, *N* = 65, 33 girls, mean age 56 months, range 51–62; a 5- to 6-year-old group, *N* = 26, 14 girls, mean age 69, range 63–74; and a 6- to 7-year-old group, *N* = 55, 33 girls, mean age 79, range 75–86. As there were far fewer children in the 5- to 6-year-old class we used a median split to divide children in to a younger group, *N* = 73, 39 girls, mean age 57, range 51–65, and an older group, *N* = 73, 41 girls, mean age 78, range 66–86. Children were recruited from and tested at a school serving a predominantly working-class white population in the UK.

#### Materials

We used the original tube (22 cm tall with 4 cm opening) with a round green bucket, and cartoon character stickers. On the pipecleaner version of the task children were presented with a 29 cm white pipecleaner and a 29 cm piece of black string. In the dowel version of the task children were presented with a 28 cm long piece of dowel (diameter = 1.5 cm, the dowel had three holes drilled in to it, one in the middle and one 1 cm from each end, diameter of holes = 0.5 cm), and two shorter pieces of dowel (1 × length 10 cm, diameter = 0.5 cm; 1 × length 4 cm, diameter = 0.5 cm, see **Figure 1**).

#### Procedure

Children were tested in a quiet area away from the rest of the class by a female experimenter (SR). Children were presented twice with the same tube apparatus. On the first trial children were given either the pipecleaner and string materials or the dowel materials. They received the other set of materials for their second trial. Order of materials was counterbalanced across participants. For each trial, children were presented with the pre-baited apparatus and told ‘If you can get the sticker out of here you can keep it.’ Children were given one minute to attempt to retrieve the bucket. If they did so, they were given the sticker and progressed to the next trial. If they did not retrieve the bucket independently, the experimenter asked them to stop and put down their materials and watch her. In the pipecleaner version of the task she took an identical pipecleaner to the one available and demonstrated how to bend it into a hook. On the dowel version of the task she took an identical piece of large dowel with holes drilled in and demonstrated how to insert the 4 cm piece of dowel into one of the holes at the end of the long dowel. Children were then given 30 s to try to retrieve the sticker. As in Study 2, we used a single stage demonstration to simplify analysis and to reduce the length of time taken by the study. After the first trial, the apparatus was cleared away and reset before being re-presented to the participant with new materials. After the second version of the task, the child was thanked and returned to the classroom with his/her stickers.

### RESULTS AND DISCUSSION

In Study 3, we identified eight planned *post hoc* comparisons [Comparison of performance on the dowel and pipecleaner versions of the task separately for each age group (2), comparison between age groups’ performance on dowel and on pipecleaner tasks (2), comparison of dowel task presented first or second for each age group (2), and comparison of pipecleaner task presented first or second for each age group (2)]. We report *p*-values <0.006 as significant in our *post hoc* analyses.

Gender had no effect on children’s success (lowest *p* = 0.157) and so data from boys and girls were combined.

For the hook-making task children were coded as successful if they bent the pipecleaner into a hook and used it to retrieve the bucket within the one minute time-frame. Overall, children were poor at this task with only 20/146 succeeding at making a pipecleaner hook. Absolute success levels were relatively low on the dowel task with only 33/146 children successfully adding pieces of dowel together to make a hook tool. The dowel task was significantly easier than the pipecleaner task when we compared all age groups, McNemar, *p* = 0.041 (24 children passed only dowel and 11 passed only pipecleaner), but did not reach significance for either the older (*p* = 0.78) or younger (*p* = 0.424) age groups. There was a trend for performance on the dowel task to improve with age, χ^2^(df = 1, *N* = 146) = 5.638, *p* = 0.018, but this comparison did not reach significance for the pipecleaner task *p* = 0.092.

To explore whether children transferred their knowledge bewteen tasks we tested whether attempting a task after having seen the solution to the other task improved performance. Whether children had the hooks task first or second made no difference to their level of success, all children, χ^2^(1, *N* = 146) = 1.612, *p* = 0.204 (younger children *p* > 0.999, older *p* = 0.123). It made no difference to success levels whether children received the dowel task first or second, all children, χ^2^(1, *N* = 146) = 0.094, *p* = 0.759, (younger children *p* = 0.515, older *p* = 0.353).

We used a Fishers’ Exact Test to look for a relation between performance on the dowel and pipecleaner versions of the task. This was significant, *p* = 0.018, suggesting that the likelohood of success on the two tool-making tasks were related. However, this was largely driven by the 102 (of 146) children who failed both versions of the task (nine passed both, 24 passed only dowel, 11 passed only pipecleaner).

These results show that children did not transfer knowledge they gained from their first attempt at the task onto their second attempt. Bending a pipecleaner into a hook for oneself, or being shown how to make a hook to retrieve the bucket did not aid chidren in making a hook by adding piecing of dowel together. Similarly children who made a hook, either independently or after demonstration, by adding pieces of dowel together were not more successful at making the same tool by bending a straight pipecleaner.

## GENERAL DISCUSSION

In three studies, we sought to explore the possibility that children’s observation of others making tools may produce sufficiently robust knowledge that could be deployed after a period of time and in response to novel but similar problems. We studied young children because of recent results suggesting that tool making (as compared to tool use or non-tool action sequences) might pose particular problems for young children (Beck et al., 2011) and to see if even very young children gained robust tool-making knowledge from adult demonstrations. Broadly, we argued that to support the diversity of tools in our tool-rich culture the tool-making knowledge gained from others needs these robust qualities.

Our evidence showed that tool-making knowledge gained by observation was robust when knowledge needed to be applied to the same problem at a later time point. In Study 1, children were dramatically better at making a hook at Time 2 compared to Time 1. Four- to 6-year-olds were able to retain the knowledge they gained when, having failed to solve the task at Time 1, they saw a demonstration of how to make an appropriate tool. They reproduced the tool making they had previously seen.

In Studies 2 and 3, we turned to a different way in which tool-making knowledge could be robust: do children transfer their tool-making knowledge to new situations? In study 2, children encountered novel problems that required similar solutions, but differed in surface characteristics. Five- to 6-year-olds performed near ceiling on later trials. Younger 3- to 5-year-olds did this to some extent, succeeding on about 50% of subsequent trials, although the difference between trials was not always statistically significant. Future research should disambiguate this finding. However, in Study 3, we investigated whether children could use their knowledge of the tool required to make the same tool from a different material. Children aged 5–7 failed to transfer their tool knowledge to new materials.

In some ways our findings are unsurprising given the literature that shows that even children in the first 2 years of life can reproduce actions after time delays and in novel contexts. This was important to show in the context of tool making because it suggests that when children copy the adults’ tool-making demonstration they are learning something about the type of tool they are making, not simply copying blindly and responding the adults’ immediate actions. However, despite being able to retain tool-making knowledge over an extended period of time, children experienced some problems transferring this knowledge to related but different situations (3- to 5-year-olds in Study 2 and 4- to 7-year-olds in Study 3).

Most strikingly, even when the problem to be solved remained identical (Study 3), children did not recognize the similarity between the solutions which involved different materials and transformations (pipecleaners or dowel, Study 3). Children may have struggled to notice the similar causal structure shared between the different instantiations of the task (of the apparatus or tool-making materials). Based on the partial success by the younger children and convincing success by the older children in Study 2, it seems that children find it easier to transfer knowledge to new instantiations of the task, which share the same materials and transformation for tool making, than to transfer the abstract knowledge of the particular type of tool that is needed. It is particularly interesting that children were limited in transferring their knowledge to new tool-making materials (i.e., between dowel and pipecleaners). The exact material from which a tool is made is not critical to the solution, although, of course, the material needs to be suitable for the task in hand. This suggests an interesting limit on children’s ability to make tools to solve problems.

One possibility is that while the ability to copy and retain copied behavior is relatively early developing, transferring this knowledge to other situations relies on analogical reasoning that is later developing. While young children can attend to some relations between items from a relatively young age, it appears that changes occur in their success at drawing analogies at around 5 years of age. Rattermann and Gentner (1998) claim that this is a qualitative change: a relational shift, by which children stop focussing exclusively on surface perceptual similarity and instead move to make judgments based on relational similarity between items. Thus, is it possible that in our tasks children focus on the differences in surface features of the task and fail to notice the shared structural similarity. A different position is held by Goswami (1991) who argues that changes in children’s analogical reasoning come about because of quantitative changes in their domain knowledge. Perhaps children need to make more explicit their understanding of pliable materials and hookable objects. Our current findings do not speak to whether children’s analogical reasoning is developing or whether they are gaining greater domain specific knowledge, or indeed whether changes in the robustness of children’s tool-making knowledge is better characterized in a different way to advances in analogical reasoning.

It would be of interest to see if children’s performance on our tool-making transfer task maps onto changes in their analogical reasoning ability [which may be taken as support for Rattermann and Gentner’s (1998) position], or whether increased exposure to using hooks before attempting the tool-making task improves transfer [which would support Goswami (1991)]. Furthermore, many analogical reasoning tasks ask children explicitly to identify a target that is the same as the original. We already know that simply drawing children’s attention to the fact that they have to ‘make something’ does not help them innovate (Cutting et al., 2011). However, children may be assisted in transferring knowledge about tool making if they were directed to look for similarities between instantiations of the task. Of course, if we uncovered a need for such scaffolding, this would further underline the need for pedagogical support in children’s developing understanding of tools.

Learning about tool-making from others might offer further insights in to the nature of innovation and collaboration. It is worth considering that in our procedure children are not simply imitating the experimenter to solve the bucket/tube problem. Rather, children learn how to *make* the tool by observing the other, but they then go on to *use* it appropriately, despite not having seen such a tool used to solve this problem. In this sense the solution to the problem is reached through collaboration. Tool-making may offer a particularly rich domain in which to explore the development of collaboration as different members of a team can offer diverse insights in to the overall task. It will be interesting in the future to investigate how children collaborate to solve such problems. Similarly, one might investigate the metacognitive aspects of problem solving using such a paradigm. For example, do children attribute credit to the demonstrator who showed them how to make a tool by sharing the reward, e.g., Warneken et al. (2011), or by giving explicit credit? One reason to think that children may not explicitly recognize another’s role in problem solving comes from the wealth of literature on children’s source monitoring, where children up to at least 5 years often claim inaccurately that they found something out through their own actions, when in fact this information was provided by another (see, e.g., Robinson and Whitcombe, 2003). It is also worth noting that in our studies, the experimenter demonstrated to the children how to make an appropriate tool using ostensive cues and she did so at a point in the experiment where the child might rightly infer that she was trying to help them. It remains to be tested whether children learn tool-making information just as well from non-ostensive evidence and, separately, whether information gained in this way is as robust over time and context as that which has been explicitly taught.

These studies suggest that even in young children knowledge gained from teaching about tool-making is robust to some extent, but the ability to transfer knowledge to novel situations continues to develop in middle childhood. Following a single demonstration and experience children could replicate the same tool-making behavior after a substantial period of time. To some extent information was generalized between problems, but we speculate that development of analogical reasoning is critical for children to generalize tool-making knowledge to novel situations appropriately.

## Conflict of Interest Statement

The authors declare that the research was conducted in the absence of any commercial or financial relationships that could be construed as a potential conflict of interest.

## Footnotes

1 c.f. make tools.
